# 2-Methyl-5,6-dinitro­benzimidazolium chloride

**DOI:** 10.1107/S1600536811008105

**Published:** 2011-03-09

**Authors:** Sebla Dinçer, Gonca Gönülalan, Barış Tercan, Tuncer Hökelek

**Affiliations:** a Department of Chemistry, Ankara University, 06100 Tandoğan, Ankara, Turkey; b Department of Physics, Karabük University, 78050, Karabük, Turkey; c Department of Physics, Hacettepe University, 06800 Beytepe, Ankara, Turkey

## Abstract

In the title compound, C_8_H_7_N_4_O_4_^+^·Cl^−^, the cation possesses twofold symmetry, with the twofold axis bis­ecting the 2-methyl-5,6-dinitro­benzimidazolium cation. The methyl H atoms are disordered about this twofold axis and were assigned equal half-occupancies. The chloride anion also lies on a twofold axis. In the crystal, N—H⋯Cl and C—H⋯O hydrogen bonds link the ions to form a three-dimensional network.

## Related literature

For literature on the anti­tumour, anthelmintic, anti­bacterial, virucidal and fungicidal properties of benzimidazole derivatives, see: Refaat (2010 ▶); Laryea *et al.* (2010 ▶); Horton *et al.* (2003 ▶); Spasov *et al.* (1999 ▶); Soula & Luu-Duc (1986 ▶). For literature on the coordination and corrosion inhibitor abilities of the benzimidazoles, see: Kuznetsov & Kaza­nsky (2008 ▶); Subramanyam & Mayanna (1985 ▶). For literature on the use of benzimidazole derivatives as photographic materials and dyes, see: Hoffmann *et al.* (2011 ▶); Alamgir *et al.* (2007 ▶). For a related structure, see: Hökelek *et al.* (2002 ▶).
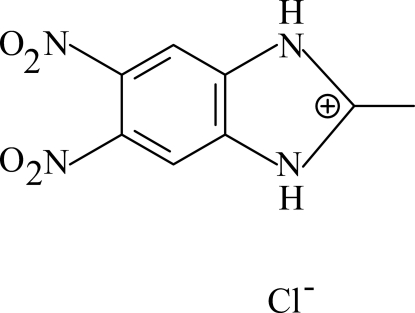


## Experimental

### 

#### Crystal data

C_8_H_7_N_4_O_4_^+^·Cl^−^
*M* *_r_* = 258.63
Orthorhombic, 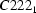

*a* = 4.9453 (1) Å
*b* = 20.4691 (4) Å
*c* = 10.4543 (3) Å
*V* = 1058.25 (4) Å^3^
*Z* = 4
Mo *K*α radiation
μ = 0.37 mm^−1^
*T* = 100 K
0.46 × 0.40 × 0.20 mm

#### Data collection

Bruker Kappa APEXII CCD area-detector diffractometer
Absorption correction: multi-scan (*SADABS*; Bruker, 2001 ▶) *T*_min_ = 0.848, *T*_max_ = 0.929
3043 measured reflections
1302 independent reflections
1264 reflections with *I* > 2σ(*I*)
*R*_int_ = 0.016

#### Refinement

*R*[*F* ^2^ > 2σ(*F* ^2^)] = 0.024
*wR*(*F* ^2^) = 0.062
*S* = 1.11
1302 reflections
83 parameters
H atoms treated by a mixture of independent and constrained refinement
Δρ_max_ = 0.27 e Å^−3^
Δρ_min_ = −0.16 e Å^−3^
Absolute structure: Flack (1983 ▶), 517 Friedel pairs
Flack parameter: 0.10 (6)

### 

Data collection: *APEX2* (Bruker, 2007 ▶); cell refinement: *SAINT* (Bruker, 2007 ▶); data reduction: *SAINT*; program(s) used to solve structure: *SHELXS97* (Sheldrick, 2008 ▶); program(s) used to refine structure: *SHELXL97* (Sheldrick, 2008 ▶); molecular graphics: *ORTEP-3 for Windows* (Farrugia, 1997 ▶) and *Mercury* (Macrae *et al.*, 2006 ▶); software used to prepare material for publication: *WinGX* (Farrugia, 1999 ▶).

## Supplementary Material

Crystal structure: contains datablocks I, global. DOI: 10.1107/S1600536811008105/su2261sup1.cif

Structure factors: contains datablocks I. DOI: 10.1107/S1600536811008105/su2261Isup2.hkl

Additional supplementary materials:  crystallographic information; 3D view; checkCIF report

## Figures and Tables

**Table 1:** Hydrogen-bond geometry (Å, °)

*D*—H⋯*A*	*D*—H	H⋯*A*	*D*⋯*A*	*D*—H⋯*A*
N2—H2*A*⋯Cl1	0.86 (2)	2.15 (2)	3.008 (1)	172.5 (18)
C2—H2⋯O1^i^	0.93	2.51	3.339 (2)	150

Symmetry code: (i) 
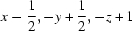
.

**Fig. 1.**
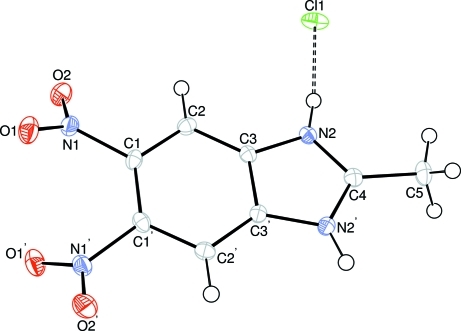
The molecular structure of the title compound with the displacement ellipsoids drawn at the 50% probability level. The N-H···Cl hydrogen bond is shown as a dashed line.

**Fig. 2.**
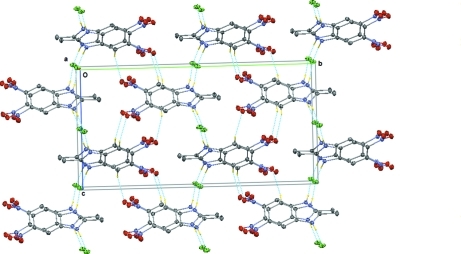
A view along the a-axis of the crystal packing of the title compound. Hydrogen bonds are shown as dashed lines [H-atoms not involved in hydrogen bonding have been omitted for clarity].

## supplementary crystallographic information

### Comment

Benzimidazole derivatives are privileged structures in pharmaceutical chemistry because of their biological activities and clinical applications. They exhibit antitumor, anthelmintic, antibacterial, virucidal and fungucidal properties (Refaat, 2010; Laryea *et al.*, 2010; Horton *et al.*, 2003; Spasov *et al.*, 1999; Soula & Luu-Duc, 1986). In addition to their biological activities, a review of the literature reveals that there are numerous studies including the coordination and corrosion inhibitor abilities of benzimidazoles (Kuznetsov & Kazansky, 2008; Subramanyam & Mayanna, 1985). Some of these derivatives, particularly nitro derivatives, are used as photographic materials in photography and on the other hand, the development of the chemistry of the benzimidazole dyes has been remarkable (Hoffmann *et al.*, 2011; Alamgir *et al.*, 2007). As a part of our ongoing investigations of benzimidazole derivatives, the title compound was synthesized and its crystal structure is reported herein.

The asymmetric unit of the title compound, (Fig. 1), contains one half of each component. It consists of an imidazole ring with the one CH_3_ and two NO_2_ groups bonded at positions 2, 5 and 6, respectively, and one chloride anion. Both the 2-methyl-5,6-dinitrobenzimidazolium moiety and the chloride anion lie on twofold axes. The methyl H atoms are disordered about the twofold axis with equal half occupancies.

In the crystal of the title compound N—H···Cl hydrogen bonds link the cations to form zigzag chains propagating in [001]. There are also C—H···O hydrogen bonds linking these chains to form a three-dimensional network (Table 1 and Fig. 2).

The crystal structure of a similar benzimidazole derivative, (C_7_H_4_N_4_O_4_).H_2_O, has been reported (Hökelek *et al.*, 2002).

### Experimental

For the preparation of the title compound a solution of 2-methyl-5-nitro- benzimidazole (3.0 g) in sulphuric acid (3.0 ml) was treated with nitric acid (6.0 ml) and refluxed for 3 h, then poured onto ice. The precipitate was filtered off and washed with cold water. Hydrogen chloride was passed into a suspension of the crude dinitro product in warm water. After cooling, the precipitate was filtered and crystallized from ethanol to give yellow block-like crystals of the the title compound (m.p. 507-512 K).

### Refinement

Atom H2A (for the NH group) was located in a difference Fourier map and was freely refined. The C-bound H-atoms were positioned geometrically with C—H = 0.93 and 0.96 Å, for aromatic and methyl H-atoms, respectively, and constrained to ride on their parent atoms, with *U*_iso_(H) = k × *U*_eq_(C), where k = 1.5 for methyl H-atoms and k = 1.2 for all other H-atoms.

### Crystal data

**Table:**

C_8_H_7_N_4_O_4_^+^·Cl^−^	*F*(000) = 528
*M**_r_* = 258.63	*D*_x_ = 1.623 Mg m^−^^3^
Orthorhombic, *C*222_1_	Mo *K*α radiation, λ = 0.71073 Å
Hall symbol: C 2c 2	Cell parameters from 2425 reflections
*a* = 4.9453 (1) Å	θ = 2.8–28.2°
*b* = 20.4691 (4) Å	µ = 0.37 mm^−^^1^
*c* = 10.4543 (3) Å	*T* = 100 K
*V* = 1058.25 (4) Å^3^	Block, yellow
*Z* = 4	0.46 × 0.40 × 0.20 mm

### Data collection

**Table:**

Bruker Kappa APEXII CCD area-detector diffractometer	1302 independent reflections
Radiation source: fine-focus sealed tube	1264 reflections with *I* > 2σ(*I*)
graphite	*R*_int_ = 0.016
φ and ω scans	θ_max_ = 28.3°, θ_min_ = 2.0°
Absorption correction: multi-scan (*SADABS*; Bruker, 2001)	*h* = −6→6
*T*_min_ = 0.848, *T*_max_ = 0.929	*k* = −20→26
3043 measured reflections	*l* = −13→13

### Refinement

**Table:**

Refinement on *F*^2^	Secondary atom site location: difference Fourier map
Least-squares matrix: full	Hydrogen site location: inferred from neighbouring sites
*R*[*F*^2^ > 2σ(*F*^2^)] = 0.024	H atoms treated by a mixture of independent and constrained refinement
*wR*(*F*^2^) = 0.062	*w* = 1/[σ^2^(*F*_o_^2^) + (0.0335*P*)^2^ + 0.4282*P*] where *P* = (*F*_o_^2^ + 2*F*_c_^2^)/3
*S* = 1.11	(Δ/σ)_max_ < 0.001
1302 reflections	Δρ_max_ = 0.27 e Å^−^^3^
83 parameters	Δρ_min_ = −0.16 e Å^−^^3^
0 restraints	Absolute structure: Flack (1983), 517 Friedel pairs
Primary atom site location: structure-invariant direct methods	Flack parameter: 0.10 (6)

### Special details

**Table:**

Geometry. All esds (except the esd in the dihedral angle between two l.s. planes) are estimated using the full covariance matrix. The cell esds are taken into account individually in the estimation of esds in distances, angles and torsion angles; correlations between esds in cell parameters are only used when they are defined by crystal symmetry. An approximate (isotropic) treatment of cell esds is used for estimating esds involving l.s. planes.
Refinement. Refinement of F^2^ against ALL reflections. The weighted R-factor wR and goodness of fit S are based on F^2^, conventional R-factors R are based on F, with F set to zero for negative F^2^. The threshold expression of F^2^ > 2sigma(F^2^) is used only for calculating R-factors(gt) etc. and is not relevant to the choice of reflections for refinement. R-factors based on F^2^ are statistically about twice as large as those based on F, and R- factors based on ALL data will be even larger.

### Fractional atomic coordinates and isotropic or equivalent isotropic displacement parameters (Å^2^)

**Table:**

	*x*	*y*	*z*	*U*_iso_*/*U*_eq_	Occ. (<1)
Cl1	0.38240 (8)	0.0000	0.5000	0.02165 (12)	
O1	0.9799 (2)	0.31462 (5)	0.62135 (10)	0.0235 (2)	
O2	0.5726 (2)	0.27552 (5)	0.60594 (10)	0.0234 (2)	
N1	0.8093 (2)	0.27227 (6)	0.64031 (11)	0.0174 (2)	
N2	0.8329 (2)	0.03251 (5)	0.68250 (10)	0.0133 (2)	
H2A	0.712 (4)	0.0200 (10)	0.628 (2)	0.035 (6)*	
C1	0.8975 (3)	0.21173 (6)	0.70323 (11)	0.0143 (2)	
C2	0.7865 (3)	0.15455 (7)	0.65679 (12)	0.0146 (3)	
H2	0.6486	0.1545	0.5963	0.018*	
C3	0.8933 (3)	0.09724 (6)	0.70596 (11)	0.0124 (2)	
C4	1.0000	−0.00475 (10)	0.7500	0.0143 (3)	
C5	1.0000	−0.07722 (10)	0.7500	0.0207 (4)	
H5A	0.8609	−0.0929	0.6937	0.031*	0.50
H5B	0.9666	−0.0929	0.8351	0.031*	0.50
H5C	1.1726	−0.0929	0.7211	0.031*	0.50

### Atomic displacement parameters (Å^2^)

**Table:**

	*U*^11^	*U*^22^	*U*^33^	*U*^12^	*U*^13^	*U*^23^
Cl1	0.01317 (19)	0.0379 (3)	0.01385 (18)	0.000	0.000	−0.00422 (19)
O1	0.0284 (5)	0.0159 (5)	0.0261 (5)	−0.0007 (4)	0.0058 (4)	0.0042 (4)
O2	0.0231 (5)	0.0213 (5)	0.0259 (5)	0.0069 (4)	−0.0046 (4)	0.0017 (4)
N1	0.0222 (6)	0.0145 (5)	0.0156 (5)	0.0036 (5)	0.0015 (4)	0.0010 (4)
N2	0.0137 (5)	0.0129 (5)	0.0133 (5)	−0.0011 (4)	0.0006 (4)	−0.0010 (4)
C1	0.0145 (6)	0.0127 (6)	0.0157 (5)	0.0026 (5)	0.0030 (5)	0.0019 (4)
C2	0.0133 (5)	0.0169 (6)	0.0137 (5)	0.0015 (5)	0.0000 (4)	0.0009 (5)
C3	0.0129 (5)	0.0125 (6)	0.0117 (5)	−0.0009 (5)	0.0026 (5)	−0.0007 (4)
C4	0.0145 (7)	0.0154 (9)	0.0131 (7)	0.000	0.0035 (6)	0.000
C5	0.0250 (10)	0.0110 (9)	0.0261 (10)	0.000	0.0025 (8)	0.000

### Geometric parameters (Å, °)

**Table:**

N1—O1	1.2259 (16)	C2—H2	0.9300
N1—O2	1.2260 (16)	C3—C3^i^	1.401 (3)
N1—C1	1.4692 (17)	C4—N2	1.3275 (16)
N2—C3	1.3801 (16)	C4—N2^i^	1.3275 (16)
N2—H2A	0.86 (2)	C4—C5	1.483 (3)
C1—C1^i^	1.409 (3)	C5—H5A	0.9600
C2—C1	1.3808 (18)	C5—H5B	0.9600
C2—C3	1.3855 (18)	C5—H5C	0.9600
			
O1—N1—O2	124.84 (12)	N2—C3—C2	131.66 (12)
O1—N1—C1	117.67 (11)	N2—C3—C3^i^	106.24 (7)
O2—N1—C1	117.41 (11)	C2—C3—C3^i^	122.08 (8)
C3—N2—H2A	123.5 (14)	N2^i^—C4—N2	109.87 (17)
C4—N2—C3	108.82 (12)	N2^i^—C4—C5	125.07 (9)
C4—N2—H2A	127.6 (14)	N2—C4—C5	125.07 (9)
C1^i^—C1—N1	121.63 (7)	C4—C5—H5A	109.5
C2—C1—N1	116.07 (11)	C4—C5—H5B	109.5
C2—C1—C1^i^	122.02 (8)	C4—C5—H5C	109.5
C1—C2—C3	115.83 (12)	H5A—C5—H5B	109.5
C1—C2—H2	122.1	H5A—C5—H5C	109.5
C3—C2—H2	122.1	H5B—C5—H5C	109.5
			
O1—N1—C1—C1^i^	33.8 (2)	C3—C2—C1—N1	172.73 (11)
O1—N1—C1—C2	−140.30 (13)	C3—C2—C1—C1^i^	−1.3 (2)
O2—N1—C1—C1^i^	−149.18 (15)	C1—C2—C3—N2	179.82 (13)
O2—N1—C1—C2	36.76 (17)	C1—C2—C3—C3^i^	−2.1 (2)
C4—N2—C3—C2	177.52 (13)	N2^i^—C4—N2—C3	0.32 (6)
C4—N2—C3—C3^i^	−0.81 (16)	C5—C4—N2—C3	−179.68 (6)

Symmetry codes: (i) −*x*+2, *y*, −*z*+3/2.

### Hydrogen-bond geometry (Å, °)

**Table:**

*D*—H···*A*	*D*—H	H···*A*	*D*···*A*	*D*—H···*A*
N2—H2A···Cl1	0.86 (2)	2.15 (2)	3.008 (1)	172.5 (18)
C2—H2···O1^ii^	0.93	2.51	3.339 (2)	150

Symmetry codes: (ii) *x*−1/2, −*y*+1/2, −*z*+1.
